# Assessment of the Quality and Reliability of Information on YouTube Regarding Angiography and Angioplasty

**DOI:** 10.7759/cureus.45885

**Published:** 2023-09-25

**Authors:** Tanya Nazar, Spurthy Namala, Rohit Rao Yelagapuri, Abhik Halder, Neera N Vora, Preyansh Doshi

**Affiliations:** 1 Internal Medicine, Government Medical College, Kozhikode, IND; 2 Internal Medicine, Gandhi Medical College, Hyderabad, IND; 3 Intensive Care Unit, Ramdev Rao Hospital, Hyderabad, IND; 4 Medicine, IQ City Medical College and Narayana Multispecialty Hospital, Durgapur, IND; 5 Internal Medicine, Gujarat Cancer Society (GCS) Medical College, Hospital and Research Centre, Ahmedabad, IND

**Keywords:** minimally invasive interventional radiology, cardiology, reliability score, global quality score, angiography and angioplasty, youtube

## Abstract

Introduction

Angiography is a method for defining the inner vessel wall and demonstrating flow through the lumen by detecting contrast injection into a blood vessel and projecting it onto a sequence of X-rays. This method is used to image the anatomical and architectural aspects of the vascular system. By employing balloon dilatation and the implantation of stents to widen the stenosed arteries, angioplasty is a form of minimally invasive endovascular treatment used to treat cardiovascular diseases and their consequences. People frequently rely on YouTube as a resource for awareness-raising and marketing activities. Animations and visual explanations can help patients understand the risks and benefits of procedures.

Aims

To assess the quality and reliability of the information on YouTube about angiography and angioplasty. We assessed quality using the GQS (Global Quality Scale) and reliability via the reliability score.

Methodology

This is an observational, cross-sectional study without the requirement of an ethics committee. It includes a questionnaire with predetermined criteria like time since upload, popularity, or type of uploader. The study assesses YouTube videos that include criteria using GQS and reliability scores. Responses recorded in Google Sheets were transferred to Microsoft Excel (Redmond, USA). Statistical analysis was performed using IBM Corp. Released 2012. IBM SPSS Statistics for Windows, Version 21.0. Armonk, NY: IBM Corp. All six authors assessed 10 YouTube videos using specific keywords. The study includes videos that meet the inclusion criteria. Videos that did not include the inclusion criteria were excluded.

Results

After applying inclusion/exclusion criteria, 57 out of 60 videos were included. Of the total videos analyzed, the majority were uploaded by various hospitals and people other than doctors and healthcare organizations. About 78.95% of the videos described the reason for angiography/plasty, followed by the anatomical area involved and the pre-procedural preparation phase. There is a significant increase in the GQS score and reliability score among the videos uploaded by doctors, hospitals, healthcare organizations, and other groups.

Conclusions

Verified health information should be uploaded responsibly by doctors, hospitals, healthcare organizations, or other agencies on social media like YouTube in a manner that is easy to understand, has a high GQS, and has a high reliability score, as it would make it simpler for the general population or viewers to have access to important health-related content they can rely on. Videos should advise the viewers to contact their doctors for all queries regarding the diagnosis or treatment of their health concerns.

## Introduction

Angiography defines the inner vessel wall and demonstrates flow through the lumen by recognizing contrast injection into a blood vessel and projecting it onto a sequence of X-rays. This method is used to image the anatomical and functional aspects of the vascular system. Angiography has developed from its initial use as a diagnostic tool to a real-time, two-dimensional presentation and three-dimensional reconstruction. The application of angiography has been broadened by improvements in imaging technology to non-invasive procedures like CT and MRI. Angiograms are invasive medical procedures. In addition to giving therapeutic choices, invasive angiography is still the gold standard for identifying most intravascular diseases. Interventional angiography gives greater-resolution imaging and is capable of detecting changes in tiny vessels [1].

By widening the stenosed arteries, angioplasty is a minimally invasive interventional technique used to treat atherosclerotic disorders and their consequences. Due to recent developments in interventional radiology, angioplasty has significantly changed since 1964, going from straightforward angioplasty with inflatable dilatation to stent implantation and atherectomy operations. The radial artery and femoral artery are the two access routes most frequently used for angioplasty [2].

At present, social media, like YouTube videos, has become a popular resource for passive and active knowledge for many people, students, and trainees for surgical procedures. Many hospitals, doctors, and healthcare organizations also upload many videos on YouTube for awareness and promotional purposes, on which people rely [3]. The animations and visual explanations can really help the patient understand the risks and benefits of the procedures and reduce anxiety levels [4,5].

Angiography has many indications, like the diagnosis and treatment of coronary artery diseases, thoracic and abdominal aortic aneurysms and dissection, aneurysms, vascular malformations, peripheral vascular diseases, hemorrhages, embolizations, cavernous-carotid fistulas, renovascular hypertension, and a few oncological causes like trans-arterial cancer therapy, renal cell carcinoma, etc. [1].

Acute coronary syndrome, coronary artery disease, disease of the carotid artery, peripheral vascular disease, renal artery stenosis, and AV fistula stenosis are just a few more indications for angioplasty [2].

The aim of this study is to assess the quality and reliability of information on YouTube regarding angiography and angioplasty. 

## Materials and methods

This is a cross-sectional type of observational study conducted on June 11, 2023. The approval of an ethical committee was not required for this study. A specific questionnaire was prepared on Google Forms, which had some predetermined criteria for characteristics of videos (time since upload, likes, comments, and type of uploader) and information circulated about angiography and angioplasty (indications, side effects, and many more). Lastly, the videos were evaluated for quality and reliability using a standard Global Quality Scale (GQS) and DISCERN score, respectively [6]. The responses were recorded in Google Sheets and transferred to Microsoft Excel (Redmond, USA). The statistical analysis was performed using IBM Corp. Released 2012. IBM SPSS Statistics for Windows, Version 21.0. Armonk, NY: IBM Corp.

All six authors assessed 10 YouTube videos using keywords like “Angioplasty”, "Angiography”, "Angioplasty procedure”, “Angiography procedure”, “Coronary Angiography”, and "Coronary Angioplasty”. Any repeated entries were deleted. The videos that satisfied all the inclusion criteria (relevant to the topic of angiography/angioplasty, in English, and with a video length of one minute to 20 minutes) were included in the study. Those videos that did not meet the inclusion criteria were excluded from the study.

## Results

A total of 60 videos were evaluated, and finally, after applying inclusions/ exclusion criteria and deleting repeats, 57 were taken into consideration.

The total number of likes was 296398, dislikes were 22093, views were 70661325, and comments were 6929. Table 1 shows the characteristics of our analyzed videos.

**Table 1:** Characteristics of YouTube videos analyzed

Criteria	n (%)
Time since uploaded	
Less than one year (< 365 days)	8 (14%)
More than one year (> 365 days)	49 (86%)
Popularity	
Total no. of views	70661325
Total no. of likes	296398
Total no. of dislikes	22093
Total no. of comments	6929
Type of uploader	
Doctor	7 (12.3%)
Hospital	23 (40.4%)
Healthcare organization	7 (12.3%)
Other	20 (35.1%)

About 80% of the videos were around a year old, and only a small percentage were published by doctors.

Out of the total videos analyzed, the majority were uploaded by various hospitals and people other than doctors and healthcare organizations. About 23 (40.4%) of the videos were uploaded by hospitals and 20 (35.1%) by others. However, only seven (12.3%) of the total analyzed videos were contributed by doctors and healthcare organizations.

Table 2 shows the type of information being circulated about the disease. About 45 (78.95%) of the videos described the reason for angiography/angioplasty, followed by the anatomical area involved and the pre-procedural preparation phase.

**Table 2:** Information about angiography and angioplasty is shared in the YouTube videos

Criteria	n (%)
Description of indications of angiography/angioplasty	25 (43.86%)
Information about investigations or tests for angina	7 (12.28%)
Information about prevention of angina	1 (1.75%)
Information about mortality	7 (12.28%)
Information about rehabilitation	10 (17.54%)
Information about support groups	0 (0%)
Information about people/patient's sharing their own experience	9 (15.79%)
Information about parent sharing their experience with their family members	1 (1.75%)
The post has a promotional content by pharmaceutical company or by doctors?	5 (8.77%)
Description of reason for angioplasty/angiography	45 (78.95%)
Description of anatomy of involved area	36 (63.16%)
Description of pre-procedural care/preparation phase	37 (64.91%)
Description of prognosis after procedure	27 (47.37%)
Description of post-procedural care	24 (42.11%)

Table 3 shows the comparison of GQS, reliability score, and video power index (VPI) based on the type of uploader. VPI is a measure of the popularity of a video based on likes and comments [6]. The videos uploaded by others and doctors had the highest median VPI of 142.15 and 140.95, respectively. The VPI was low for other uploaders, but this difference was not statistically significant (p >0.0%). The quality of information assessed by the median GQS was four for each of the doctors, hospitals, and healthcare organizations and three for others. This difference was statistically significant (p <0.04). The videos uploaded by hospitals, healthcare organizations, and doctors had higher reliability (median reliability score of 4, 4, and 3, respectively) compared to others (median reliability score of 2.5). This difference was statistically significant (p <0.05).

**Table 3:** Comparison of GQS, reliability score, and VPI based on type of uploader The data is presented in the format of median (IQ1, IQ3), where IQ stands for interquartile range. p <0.05 is significant. GQS = Global quality score; VPI: Video power index, IQ: Interquartile range

	Doctors (n=07)	Hospital (n=23)	Healthcare organization (n=07)	Other (n=20)	P-value & test used
	Median (IQ1, IQ3)	Median (IQ1, IQ3)	Median (IQ1, IQ3)	Median (IQ1, IQ3)	Test Used: Kruskal-Wallis Test
VPI	140.95 (27.86, 866.61)	117.26 (3.87, 429.39)	39.6 (13.76, 67.81)	142.15 (5.61, 359.75)	P-value = 0.714
GQS	4 (3, 5)	4 (3, 4)	4 (4, 5)	3 (2, 4)	P-value = 0.042
Reliability Score	3 (3, 4)	4 (3, 4)	4 (3, 5)	2.5 (2, 3.75)	P-value = 0.010

## Discussion

The widespread availability and popularity of online platforms have transformed the nature of medical information distribution in recent years. Among these mediums, YouTube has emerged as a popular destination for people seeking health-related information. We explore YouTube's medical information ecosystem in this study, analyzing a varied range of videos involving angina and angioplasty in order to gain deeper insights regarding the content, uploader dynamics, and quality indicators of these health-related videos.

On YouTube, we evaluated 57 videos. Most videos available for more than a year (86%) suggest that YouTube serves as an archive of previous angina and angioplasty knowledge. This long-lasting material indicates the continuing importance of specific medical concepts and helps to educate users. Overall, old videos on YouTube contribute to a better understanding of medical milestones and the evolution of healthcare practices over time. Viewer interaction and interest are reflected in the distribution of likes, dislikes, and comments. Although the number of likes (296,398) significantly outweighs the number of dislikes (22,093), showing a broad positive attitude, the comments (6,929) indicate strong audience participation and conversations about these medical concerns. In a study conducted by Satinder et al. [7], it was observed that the average number of likes was highest for paramedic videos (6,788.8). This highlights YouTube's potential as a medium for health-related discussion and information exchange.

A significant portion of videos (43.86%) focus on outlining the indications for angiography and angioplasty, indicating a primary emphasis on proving the necessity of the medical treatments. In a study conducted by Byeong et al. [8], items of “indications” were found to be particularly low. Further investigation demonstrates that a considerable percentage of videos (78.95%) address the reasons for undergoing angioplasty or angiography, giving viewers insight into the medical conditions that justify these procedures. These percentages represent 25 and 45 videos, respectively, emphasizing the prevalence of procedural explanations and clinical reasoning in the content analyzed. In contrast, there is a significant gap in preventative and vaccine material (1.75%), which is critical for public health education. The fact that this topic is only represented in one video out of 57 indicates a possible area for improvement, emphasizing the need for more thorough coverage of preventive measures in future health-related videos. In contrast, in a study conducted by Ignacio et al. [9], 59% of the videos discussed the benefits of the vaccine, and 39% of the videos discussed the adverse effects. Rehabilitation (17.54%) and patient experiences (15.79%) videos provide a well-rounded approach to medical information distribution. Similarly, only 21% of the videos in Tomasz’s study feature patient experience [10]. The proportions are 10 and 9, respectively, showing a notable emphasis on patient-centered content that goes beyond clinical processes to cover holistic aspects of healthcare and well-being [11].

It is worth noting that no video’ in the dataset mention support groups, which are an important resource for patients seeking social and emotional assistance. This omission (0%) highlights a previously undiscovered component of medical information on YouTube, indicating an opportunity to transform video that builds a sense of community and shared experiences among patients. Furthermore, the inclusion of promotional content by pharmaceutical firms or doctors (8.77%) highlights the various traits of content on YouTube. These five promotional videos highlight the platform’s function in not only transmitting educational content but also promoting commercial interactions in the medical field. More diverse content can better fulfill the varying informational needs and interests of viewers seeking information on angina and angioplasty.

Videos posted by doctors have a median GQS of 4, indicating good conformity with established medical principles. Their median reliability score of 3 indicates moderate reliability, while their median VPI of 140.95 indicates that viewers find these videos to be fairly informative and effective. Similarly, a study by Leva et al. [12] found that dental professionals who produced higher-quality videos had higher means of GQS [P =.035]. Similarly, hospital-uploaded videos show a GQS of 4, indicating adherence to guidelines. The greater reliability score (median of 4) increases their trustworthiness, although a somewhat lower VPI (117.26) indicates that viewers find these videos useful but maybe less compelling than doctor-uploaded content. In contrast to the study by Bakshi, which evaluated YouTube as a reliable source for patient education on aortic valve stenosis, videos submitted by non-professional sources received more views than those uploaded by professional sources. [10] Healthcare organization videos consistently have a GQS of 4, indicating their dedication to guideline-based content. Their comparable reliability score of 4 highlights their dependability. The significantly lower VPI (39.6), on the other hand, indicates that viewers may find these videos less impactful, maybe due to variations in presentation or viewer interaction tactics. Interestingly, videos from other sources have a somewhat lower GQS [10], indicating modest compliance with the rules. Their reliability score (median of 2.5) implies that they are credible. Regardless of these measurements, the greater VPI (142.15) implies that viewers regard these videos as very helpful and influential, maybe due to captivating presentation styles or relatable content. In a similar study by Tolga et al. [13], YouTube videos uploaded by laypersons are of poor quality, although these videos have higher rates of likes and VPI values. These findings shed light on the dynamic link between YouTube content quality, dependability, and audience perception. The GQS and reliability scores provide information about guideline adherence and trustworthiness, while the VPI represents viewers perceived value. The diversity of uploader types emphasizes the importance of an inclusive approach, where both adherence to guidelines and effective communication are required. This comprehensive viewpoint has the potential to improve the quality and impact of medical information transmission while also promoting trust and understanding within the YouTube health information ecosystem [14,15].

Our study's limitations include solely evaluating online content on YouTube and not covering other social media sites. Our study's methodology and approach focused on evaluating presently available web information without considering the impact on viewers or patients. In addition, our research looked at YouTube videos throughout a certain period. YouTube is a dynamic video-sharing network with constantly changing content. Furthermore, audience interaction with the videos was analyzed just on YouTube.com and excluded YouTube videos posted on other websites or social media platforms. Interobserver variance is likely. All of the videos specified were solely in English and Hindi.

Limitations

One of the drawbacks of our study is that we only evaluated YouTube-based online content. Other social media platforms were not examined. Without considering the effect on viewers or patients, this study's method and strategy concentrated on assessing already accessible web information. This study also examined YouTube videos over a specific time frame. YouTube, a dynamic video-sharing network, has content that is always changing. Additionally, YouTube videos broadcast on different websites or social media platforms were not included in the analysis of audience interaction with the videos, which was limited to YouTube.com. Variation between observers is likely. The only languages available for the videos were English and Hindi.

## Conclusions

Verified health information should be uploaded responsibly by doctors, hospitals, healthcare organizations, or other agencies on social media like YouTube in a manner that is easy to understand, has a high GQS, and has a high reliability score, as it would make it simpler for the general population or viewers to have access to important health-related content they can rely on. Also, the video should advise the viewers to contact their doctors for all queries regarding the diagnosis or treatment of their health concerns, as self-diagnosing and self-treating themselves can prove to be harmful.
